# An In Vivo Magnetic Resonance Spectroscopy Study of the Effects of Caloric and Non-Caloric Sweeteners on Liver Lipid Metabolism in Rats

**DOI:** 10.3390/nu9050476

**Published:** 2017-05-10

**Authors:** Sharon Janssens, Jolita Ciapaite, Justina C. Wolters, Natal A. van Riel, Klaas Nicolay, Jeanine J. Prompers

**Affiliations:** 1 Biomedical Nuclear Magnetic Resonance (NMR), Department of Biomedical Engineering, Eindhoven University of Technology, P.O. Box 513, 5600 MB Eindhoven, The Netherlands; janssenssharon@gmail.com; 2 Center for Liver, Digestive and Metabolic Diseases, Department of Pediatrics, University of Groningen, University Medical Center Groningen, Hanzeplein 1, 9713 GZ Groningen, The Netherlands; j.ciapaite@umcg.nl; 3 Systems Biology Centre for Energy Metabolism and Ageing, University of Groningen, University Medical Centre Groningen, Hanzeplein 1, 9713 GZ Groningen, The Netherlands; justina.c.wolters@rug.nl; 4 Department of Pharmacy, Analytical Biochemistry, University of Groningen, Antonius Deusinglaan, 9713 AV Groningen, The Netherlands; 5 Computational Biology, Department of Biomedical Engineering, Eindhoven University of Technology, P.O. Box 513, 5600 MB Eindhoven, The Netherlands; n.a.w.v.riel@tue.nl

**Keywords:** obesity, fatty liver disease, hepatic steatosis, carbohydrate, glucose, fructose, aspartame

## Abstract

We aimed to elucidate the effects of caloric and non-caloric sweeteners on liver lipid metabolism in rats using in vivo magnetic resonance spectroscopy (MRS) and to determine their roles in the development of liver steatosis. Wistar rats received normal chow and either normal drinking water, or solutions containing 13% (*w*/*v*) glucose, 13% fructose, or 0.4% aspartame. After 7 weeks, in vivo hepatic dietary lipid uptake and de novo lipogenesis were assessed with proton-observed, carbon-13-edited MRS combined with ^13^C-labeled lipids and ^13^C-labeled glucose, respectively. The molecular basis of alterations in hepatic liver metabolism was analyzed in detail ex vivo using immunoblotting and targeted quantitative proteomics. Both glucose and fructose feeding increased adiposity, but only fructose induced hepatic lipid accumulation. In vivo MRS showed that this was not caused by increased hepatic uptake of dietary lipids, but could be attributed to an increase in de novo lipogenesis. Stimulation of lipogenesis by fructose was confirmed by a strong upregulation of lipogenic enzymes, which was more potent than with glucose. The non-caloric sweetener aspartame did not significantly affect liver lipid content or metabolism. In conclusion, liquid fructose more severely affected liver lipid metabolism in rats than glucose, while aspartame had no effect.

## 1. Introduction

In 2013, an estimated 2.1 billion people were overweight or obese compared with 857 million people in 1980 [1]. The rise in obesity is associated with an increased prevalence of non-alcoholic fatty liver disease (NAFLD), which can progress into non-alcoholic steatohepatitis (NASH), liver cirrhosis, and hepatocellular carcinoma [2,3,4]. The increased consumption of simple carbohydrates has been identified as one of the contributing factors to the obesity epidemic [5]. Especially the chronic consumption of fructose, as opposed to glucose, has been linked to the development of obesity, insulin resistance, dyslipidemia, and hepatic steatosis [6,7,8,9,10].

Unlike glucose, fructose is mainly metabolized by the liver. The metabolism of fructose to triose phosphate bypasses phosphofructokinase, a key regulatory step of glycolysis, allowing unregulated entry of fructose into glycolysis independent of hepatic energy status. This leads to an excess production of triose phosphates, which serve as precursors for de novo lipogenesis [9,11,12]. Additionally, fructose consumption causes activation of transcription factors regulating de novo lipogenesis, i.e., sterol regulatory element-binding protein-1c (SREBP-1c) and carbohydrate-responsive element-binding protein (ChREBP), resulting in increased expression of lipogenic enzymes such as fatty acid synthase (FAS) and acetyl-CoA carboxylase (ACC) [9,10,11,13,14]. ACC catalyzes the conversion of acetyl-CoA into malonyl-CoA, providing the building blocks for fatty acid synthesis. At the same time, increased levels of malonyl-CoA as a result of fructose-induced overexpression of ACC suppresses fatty acid β-oxidation through inhibition of carnitine palmitoyl transferase 1 (CPT1) [15]. In addition, fructose suppresses the oxidation of fatty acids by decreasing the activity of peroxisome proliferator-activated receptor α (PPARα) [14,16,17,18]. Thus, the stimulation of de novo lipogenesis and the inhibition of fatty acid β-oxidation likely contribute to fructose-induced hepatic lipid accumulation. Moreover, fructose feeding has been shown to increase the intestinal production of apolipoprotein B48 (apoB48) [19,20,21], which may promote the absorption of dietary lipids from the intestine, resulting in an increased influx of diet-derived lipids into the liver. However, the relative importance of the different pathways leading to fructose-induced hepatic lipid accumulation remains unknown.

Non-caloric sweeteners simulate the sweet taste of sugars without the calories and are of great value in the fight against obesity [22]. Some studies showed beneficial effects of the consumption of non-caloric sweeteners by inducing weight loss and reducing the risk factors for metabolic syndrome [23,24,25]. In contrast, others demonstrated a correlation between the use of non-caloric sweeteners and weight gain and increased risk of type 2 diabetes and insulin resistance [26,27,28,29]. The most controversial and most used non-caloric sweetener is aspartame (methyl l-α-aspartyl-l-phenylalaninate). Aspartame consumption has been shown to increase fasting blood glucose levels and induce glucose intolerance in rats and mice, and these effects have been attributed to compositional and functional changes in gut microbiota [26,30,31]. However, data on the effects of aspartame on hepatic lipid metabolism are greatly lacking.

The aim of this study was to investigate the effects of caloric (glucose and fructose) and non-caloric (aspartame) sweeteners on liver lipid content and metabolism in vivo in rats. We recently developed a method that combines localized proton-observed, carbon-13-edited magnetic resonance spectroscopy (^1^H-[^13^C] MRS) with the oral administration of ^13^C-labeled lipids to determine dietary lipid uptake in vivo [32,33]. Here we introduce a new variant of this approach, in which we administer ^13^C-labeled glucose instead of ^13^C-labeled lipids. In this case, the ^13^C-labeled liver lipids detected by ^1^H-[^13^C] MRS originate from the conversion of ^13^C-labeled glucose to ^13^C-labeled lipids through de novo synthesis. This novel application of ^1^H-[^13^C] MRS with the oral administration of ^13^C-labeled glucose thus allows in vivo assessment of the contribution of de novo lipogenesis to hepatic lipid accumulation. In the present study, we applied both methods, i.e., ^1^H-[^13^C] MRS with the administration of ^13^C-labeled lipids and ^1^H-[^13^C] MRS with the administration of ^13^C-labeled glucose, to determine in vivo dietary lipid uptake and de novo lipogenesis, respectively, in the livers of rats receiving either normal drinking water, or 13% (*w*/*v*) glucose, 13% (*w*/*v*) fructose, or 0.4% (*w*/*v*) aspartame in their drinking water, for a period of 7 weeks. The molecular basis of alterations in hepatic liver metabolism was analyzed in detail ex vivo using immunoblotting and targeted quantitative proteomics.

## 2. Materials and Methods

### 2.1. Animals and Diets

Adult male Wistar rats (350 ± 2 g, 10–11 weeks of age, *n* = 60; Charles River Laboratories, The Netherlands) were housed in pairs in individually ventilated cages with corn cob bedding and standard cage enrichment at 20 °C and 50% humidity on a 12 h light-dark cycle. All animals received normal chow (9 energy percent (En%) from fat, 67 En% from carbohydrates, 24 En% from protein; R/M-H diet, Ssniff Spezialdiäten GmbH, Soest, Germany) for the duration of the study. After one week of acclimatization, the rats were divided into four groups receiving either normal drinking water (CON), or 13% (*w*/*v*) glucose (GLU), 13% (*w*/*v*) fructose (FRUC), or 0.4% (*w*/*v*) aspartame (ASP) in their drinking water. The animals had ad libitum access to food and liquids. Body weight and food and drink intake were determined weekly. The rats received the diets for a period of 7 weeks, after which each dietary group was divided into two subgroups: experimental group 1 (*n* = 9 per diet group) for MRS measurements to determine dietary lipid uptake and for an oral glucose tolerance test (OGTT), and experimental group 2 (*n* = 6 per diet group) for MRS to determine de novo lipogenesis and for hepatic biochemical analyses. Blood samples were taken from the vena saphena before each MRS experiment and were collected in paraoxon-coated capillaries to prevent lipolysis. The samples were centrifuged at 1000× *g* for 10 min and plasma was frozen in liquid nitrogen and stored at −80 °C for later analysis. All animal experiments were reviewed and approved by the Animal Ethics Committee of Maastricht University (DEC-UM, Maastricht, The Netherlands; project number: 2013-011; date of approval: 20 March 2013).

### 2.2. MRS Experiments

To determine total (^12^C + ^13^C) intrahepatocellular lipid (IHCL) content and natural abundance ^13^C enrichment of IHCL in liver after 7 weeks of diet, all animals (*n* = 15 per diet group) were subjected to baseline ^1^H-[^13^C] MRS measurements in a fed condition.

Two days later, rats from experimental group 1 received 1.5 g [U-^13^C] labeled algal lipid mixture (^13^C enrichment > 98%; fatty acid composition: 53% palmitic acid, 9% palmitoleic acid, 28% oleic acid, and 6% linoleic acid; Buchem B.V., Apeldoorn, The Netherlands) per kg body weight by oral gavage. The following 4 h the rats remained fasted, after which ^1^H-[^13^C] MRS experiments were performed to determine ^13^C-enriched IHCL concentrations.

Starting on the day after baseline MRS measurements, rats from experimental group 2 were administered 3.33 g [U-^13^C_6_]glucose (^13^C enrichment >98%; Buchem B.V., Apeldoorn, The Netherlands) per kg body weight by oral gavage, two times a day for a period of 5 days. The following day, ^1^H-[^13^C] MRS experiments were performed in a fed condition to determine ^13^C-enriched IHCL concentrations. After these MRS measurements, animals from experimental group 2 were euthanized by incising the vena cava inferior, and the median lobe of the liver was excised and stored at −80 °C.

^1^H-[^13^C] MRS experiments were performed on a 7 T horizontal Bruker MR system (Bruker, Ettlingen, Germany), as described previously [32]. During the MRS experiments, animals were anaesthetized using 1.5–2.5% isoflurane (IsoFlo^®^; Abbott Laboratories Ltd., Maidenhead, Berkshire, UK). Total (^12^C + ^13^C) and ^13^C-labeled IHCL levels are presented as a percentage of the unsuppressed water signal measured in the same voxel. The relative ^13^C enrichment determined at baseline was used to correct the ^13^C-enriched IHCL levels after administration of ^13^C-labeled lipids or glucose for natural abundance of ^13^C.

### 2.3. Oral Glucose Tolerance Test

In experimental group 1, two days after the last MRS measurements, an OGTT was performed (*n* = 9 per diet group). After an overnight fast, rats received 1 mg/g body weight glucose orally. Blood samples were taken from the vena saphena just before and at 15, 30, 45, 60, 90, and 120 min after glucose administration. Plasma glucose concentration was determined using a HemoCue Glucose 201 RT Analyzer (HemoCue AB, Ängelholm, Sweden), while plasma insulin concentration was analyzed using the Rat Insulin ELISA kit (Mercodia, Uppsala, Sweden). Areas under the glucose (AUC_g_) and insulin (AUC_i_) curves were calculated. Directly after the OGTT, animals from experimental group 1 were euthanized by incising the vena cava inferior, and the median lobe of the liver was excised and stored at −80 °C.

### 2.4. Plasma and Tissue Analyses

Plasma triglyceride (TG) and alanine aminotransferase (ALT) were determined using serum TG determination kit (Sigma-Aldrich, Zwijndrecht, The Netherlands) and EnzyChrom™ ALT assay kit (Bio-Connect Diagnostics, Huissen, The Netherlands), respectively, following the manufacturer’s instructions. Liver malonyl-CoA content was determined as described in [34].

### 2.5. Determination of Glycogen Content in Liver

A 200-mg snap-frozen liver sample was homogenized in 10 mL of 25 mM citrate solution (pH 4.2) containing 2.5 g/L NaF and centrifuged at 10,000× *g* for 8 min to remove debris. Glycogen content in the supernatant was determined using EnzyChrom™ glycogen assay kit (Bio-Connect Diagnostics, Huissen, The Netherlands) according to the manufacturer’s instructions.

### 2.6. Glycolytic Enzyme Activities

Pieces of snap-frozen liver were powdered in liquid nitrogen and 10% (*w*/*v*) homogenates were prepared in ice cold phosphate-buffered saline (PBS), pH 7.4. Homogenates were sonicated for 30 s in the pulse mode (pulse duration 1 s, interval between the pulses 1 s, amplitude 20%) on ice, followed by 10 min centrifugation at 1000× *g*, 4 °C. The supernatant was used for spectrophotometric determination of enzyme activities of phosphoglucose-isomerase [35], phosphoglycerate kinase [36], and pyruvate kinase [37]. Protein concentrations in the supernatants were determined using the bicinchoninic acid (BCA) protein assay kit (Pierce, Thermo Fisher Scientific Inc., Rockford, IL, USA) and enzyme activities were expressed per mg liver protein.

### 2.7. Western Blot Analysis

Protein expression levels of ACC, FAS, SREBP-1c, ChREBP, and PPARα were determined by immunoblotting as described in the Supporting Information.

### 2.8. Targeted Quantitative Mitochondrial Proteomics

Selected 47 mitochondrial proteins involved in substrate transport, oxidative phosphorylation, fatty acid β-oxidation, and tricarboxylic acid (TCA) cycle were quantified in liver samples using targeted quantitative proteomics as described in [38].

### 2.9. Statistical Analysis

Data are expressed as means ± standard error of the mean (SEM). The statistical significance of differences among the diet groups was assessed using one-way analysis of variance (ANOVA) with Tukey honest significant difference (HSD) post-hoc analyses. Differences between the two experimental groups were determined by univariate ANOVA with Tukey HSD post-hoc analyses. Statistical analyses were performed using IBM SPSS statistics 22 software package (SPSS, Inc.; Chicago, IL, USA). The level of significance was set at *p* < 0.05.

## 3. Results

### 3.1. Caloric Sweeteners Increase Adiposity without an Effect on Body Weight

**Figure 1:**
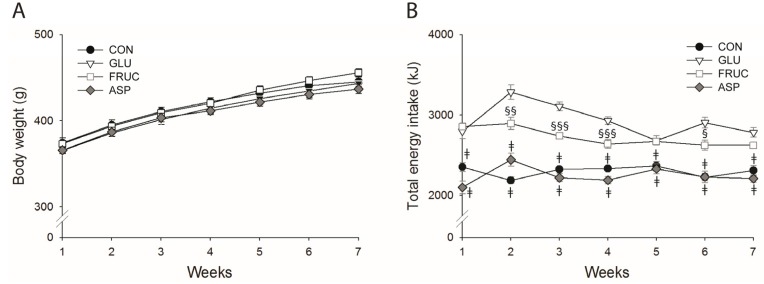
Body weight (**A**) and total energy intake (**B**) of rats receiving normal water (CON), a 13% (*w*/*v*) glucose solution (GLU), a 13% (*w*/*v*) fructose solution (FRUC), or a 0.4% (*w*/*v*) aspartame solution (ASP), determined weekly (*n* = 15 per diet group). Data are expressed as means ± standard error of the mean (SEM). ǂ *p* < 0.001 vs. GLU and FRUC; ^§^ *p* < 0.05, ^§§^ *p* < 0.01, ^§§§^ *p* < 0.001 vs. GLU.

Before the start of the diets, body weights were not significantly different between groups (Table 1). During the diet period, body weights increased similarly among the different groups (Figure 1A) and after 7 weeks of diet body weights were not significantly different (Table 1). However, both epididymal and perirenal fat pads of rats in the caloric sweetener groups GLU and FRUC were increased compared with rats in the CON and ASP groups (Table 1). Food intake was lower in the GLU and FRUC groups compared with CON and ASP, but their drink intake was higher, resulting in a higher total energy intake in GLU and FRUC compared with CON and ASP groups (Figure 1B, Table 1). Furthermore, animals in the FRUC group consumed more food, but had a lower drink intake compared with GLU animals, resulting in a slightly lower total energy intake in FRUC compared with GLU.

In the FRUC group, plasma TG concentrations were higher compared with the ASP group (Table 1). Moreover, plasma ALT levels were higher in FRUC animals compared with CON and GLU animals (Table 1).

Liver weight and liver glycogen concentrations were analyzed separately per experimental group (Table 1), because liver tissue was harvested under different conditions, i.e., fasted versus fed. In both experimental groups, FRUC animals had higher liver weights compared with GLU and ASP, while there were no significant differences in liver glycogen content between the diet groups. When comparing between the two experimental groups, liver weight and liver glycogen content were higher in experimental group 2 compared with experimental group 1, which is likely explained by the fasted state of animals in experimental group 1.

### 3.2. Both Caloric and Non-Caloric Sweeteners Affect Whole-Body Glucose Homeostasis

Whole-body glucose tolerance was assessed with an OGTT (Table 2). There were no significant differences in fasting plasma glucose or AUC_g_ among the different diet groups. Fasting plasma insulin, on the other hand, was higher in ASP and tended to be higher in GLU (*p* = 0.051) and FRUC (*p* = 0.127) compared with CON. AUC_i_ and the product of AUC_g_ and AUC_i_ also tended to be increased in GLU, FRUC, and ASP compared with CON, although this only reached statistical significance in the case of FRUC.

### 3.3. Fructose Stimulates Hepatic De Novo Lipogenesis

**Figure 2:**
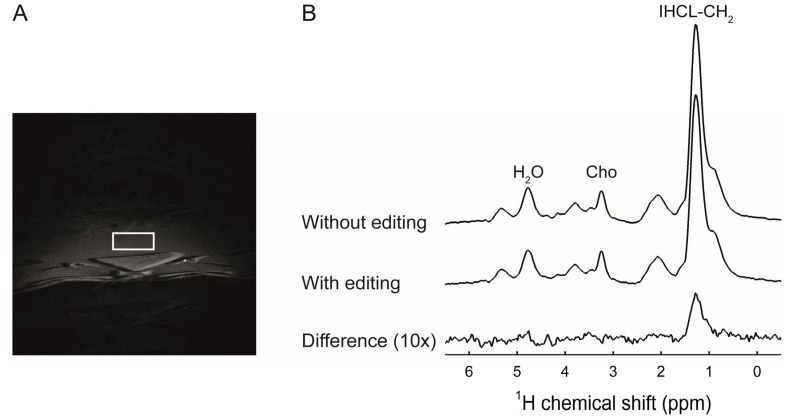
(**A**) T_1_-weighted transversal image of the abdomen of a rat receiving a 13% (*w*/*v*) fructose solution. The positioning of a 5 × 2 × 4 mm^3^ voxel in the median lobe of the liver for ^1^H-[^13^C] magnetic resonance spectroscopy (MRS) is indicated by the white square; (**B**) ^1^H-[^13^C] MRS spectra from the voxel in panel A. Spectra were acquired after 5 days of [U-^13^C_6_]glucose administration. Spectra without ^13^C editing, with ^13^C editing, and the calculated difference spectrum containing only ^13^C-coupled ^1^H resonances (10× magnification) are shown. Peak annotations: Cho, choline; IHCL, intrahepatocellular lipids. Total IHCL content was quantified from the spectrum without ^13^C editing and ^13^C-enriched IHCL content was determined from the difference spectrum.

**Figure 3:**
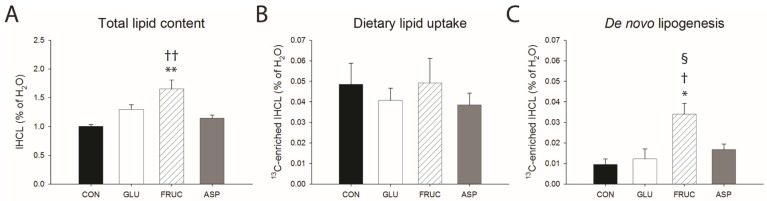
(**A**) Total lipid content at baseline (*n* = 15 per diet group); (**B**) ^13^C-enriched IHCL 4 h after the oral administration of [U-^13^C] algal lipid mixture (dietary lipid uptake; *n* = 9 per diet group); and (**C**) ^13^C-enriched IHCL after 5 days of oral administration of [U-^13^C_6_]glucose (de novo lipogenesis; *n* = 6 per diet group), in rats receiving normal water (CON), a 13% (*w*/*v*) glucose solution (GLU), a 13% (*w*/*v*) fructose solution (FRUC), or a 0.4% (*w*/*v*) aspartame solution. Data are expressed as means ± SEM. * *p* < 0.01, ** *p* < 0.001 vs. CON; ^†^ *p* < 0.05, ^††^ *p* < 0.01 vs. ASP; ^§^ *p* < 0.05 vs. GLU.

Next, we assessed the effects of caloric and non-caloric sweeteners on hepatic lipid metabolism in vivo using ^1^H-[^13^C] MRS (Figure 2). Figure 3 shows total IHCL content and ^13^C-enriched IHCL content after the administration of [U-^13^C] algal lipid mixture or [U-^13^C_6_]glucose. After 7 weeks of diet, IHCL levels were significantly higher in FRUC animals compared with CON and ASP (Figure 3A). Four hours after the administration of [U-^13^C] algal lipid mixture, levels of ^13^C-enriched IHCL were not significantly different among the groups, showing that dietary lipid uptake in the liver was not significantly affected by glucose, fructose, or aspartame consumption (Figure 3B). After 5 days of [U-^13^C_6_]glucose administration, on the other hand, ^13^C-enriched IHCL was significantly higher in FRUC compared with all other groups, demonstrating increased de novo lipogenesis in the liver upon fructose feeding (Figure 3C).

**Figure 4:**
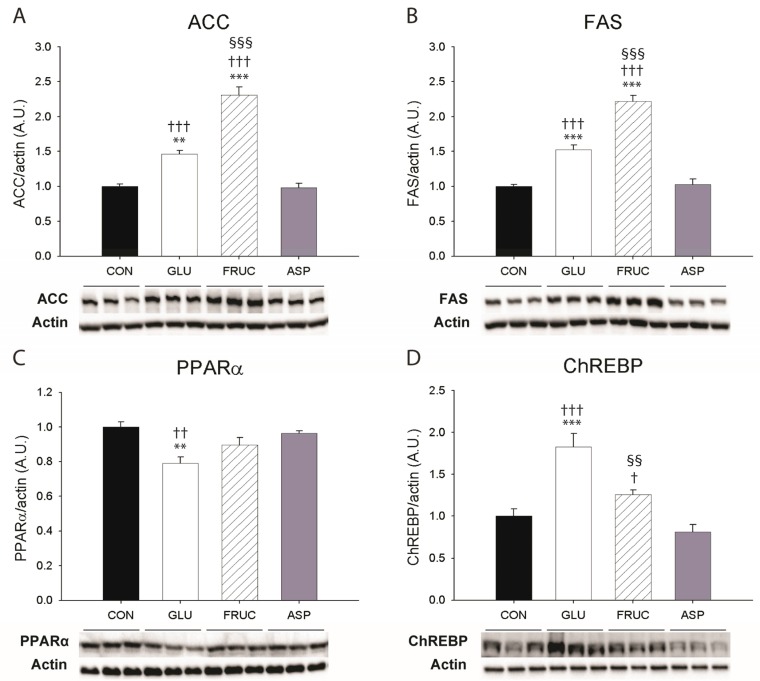

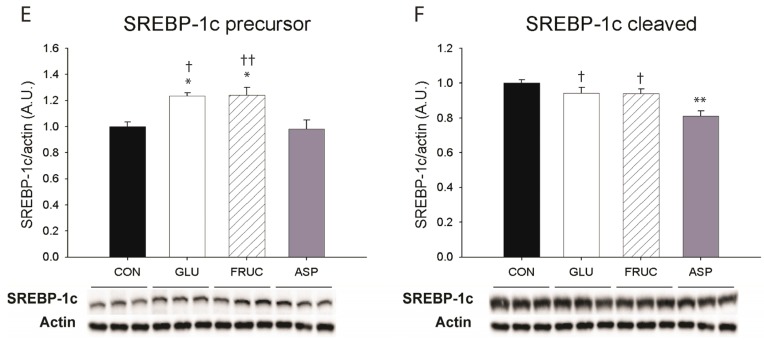
Protein expression levels of (**A**) acetyl-CoA carboxylase (ACC); (**B**) fatty acid synthase (FAS); (**C**) peroxisome proliferator-activated receptor α (PPARα); (**D**) carbohydrate-responsive element-binding protein (ChREBP); (**E**) 128 kDa precursor sterol regulatory element-binding protein-1c (SREBP-1c); and (**F**) 65 kDa cleaved SREBP-1c, in livers of rats receiving normal water (CON), a 13% (*w*/*v*) glucose solution (GLU), a 13% (*w*/*v*) fructose solution (FRUC), or a 0.4% (*w*/*v*) aspartame solution (*n* = 6 per diet group). All data were normalized to β-actin expression levels and are expressed relative to the controls (CON). Data are expressed as means ± SEM. * *p* < 0.05, ** *p* < 0.01, *** *p* < 0.001 vs. CON; ^†^ *p* < 0.05, ^††^ *p* < 0.01, ^†††^ *p* < 0.001 vs. ASP; ^§§^ *p* < 0.01, ^§§§^ *p* < 0.001 vs. GLU.

**Figure 5:**
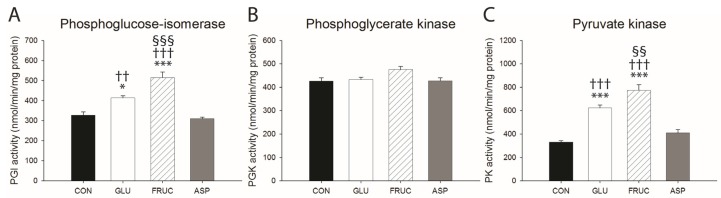
Glycolytic enzyme activities in liver. Activity of (**A**) phosphoglucose-isomerase (PGI); (**B**) phosphoglycerate kinase (PGK) and (**C**) pyruvate kinase (PK), in livers of rats receiving normal water (CON), a 13% (*w*/*v*) glucose solution (GLU), a 13% (*w*/*v*) fructose solution (FRUC), or a 0.4% (*w*/*v*) aspartame solution (*n* = 6 per diet group). Data are expressed as means ± SEM. * *p* < 0.05, *** *p* < 0.001 vs. CON; ^††^ *p* < 0.01, ^†††^ *p* < 0.001 vs. ASP; ^§§^ *p* < 0.01, ^§§§^ *p* < 0.001 vs. GLU.

Protein expression levels of key lipogenic enzymes ACC and FAS were not affected in the ASP group, but were increased in livers from the GLU and FRUC groups compared with the CON group, and the effect was stronger in FRUC compared with GLU (Figure 4A,B). Furthermore, fructose but not glucose feeding significantly increased the hepatic concentration of the ACC product malonyl-CoA compared to the CON group (Table 1). Total hepatic protein levels of SREBP-1c were only mildly affected in livers of GLU and FRUC animals, as indicated by a slight upregulation of SREBP-1c precursor compared to the CON group without an effect on cleaved SREBP-1c (Figure 4E,F). Aspartame feeding resulted in lower protein levels of cleaved SREBP-1c compared to all other experimental groups. Total protein levels of ChREBP were increased in response to glucose and fructose but not aspartame feeding compared to the CON group (Figure 4D). The strongest induction was observed in the GLU group. Upregulation of ChREBP protein levels was accompanied by increased activities of glycolytic enzymes phosphoglucose-isomerase (Figure 5A) and, even more notably, pyruvate kinase (Figure 5C) in livers from GLU and FRUC animals, with the strongest effects observed in the FRUC group. The enzyme activity of phosphoglycerate kinase (Figure 5B) tended to be higher in FRUC compared with CON (*p* = 0.056). The activities of glycolytic enzymes were not significantly affected by aspartame feeding. The protein levels of PPARα were slightly decreased in GLU but not FRUC and ASP groups compared with CON (Figure 4C).

### 3.4. Proteins Involved in Mitochondrial Oxidative Metabolism Are Differentially Affected by Glucose and Fructose Feeding

**Figure 6:**
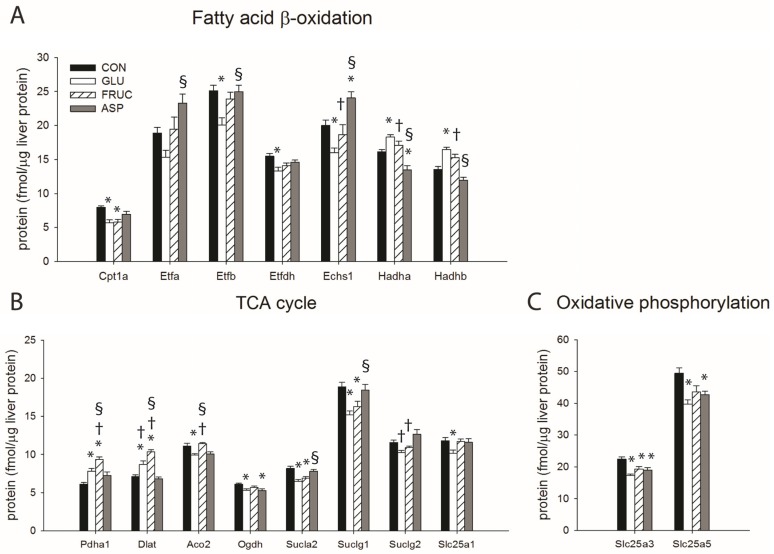
Targeted quantitative mitochondrial proteomics of proteins involved in (**A**) fatty acid β-oxidation; (**B**) tricarboxylic acid (TCA) cycle; and (**C**) oxidative phosphorylation, in livers of rats receiving normal water (CON), a 13% (*w*/*v*) glucose solution (GLU), a 13% (*w*/*v*) fructose solution (FRUC), or a 0.4% (*w*/*v*) aspartame solution (*n* = 6 per diet group). Gene names and corresponding protein names: Cpt1a: Carnitine O-palmitoyltransferase 1A; Etfa: Electron-transfer-flavoprotein, alpha polypeptide; Etfb: Electron-transfer-flavoprotein, beta polypeptide; Etfdh: Electron-transferring-flavoprotein dehydrogenase; Echs1: Enoyl CoA hydratase, short chain, 1; Hadha: Hydroxyacyl-CoA dehydrogenase/3-ketoacyl-CoA thiolase/enoyl-CoA hydratase (trifunctional protein), alpha subunit; Hadhb: Hydroxyacyl-CoA dehydrogenase/3-ketoacyl-CoA thiolase/enoyl-CoA hydratase (trifunctional protein), beta subunit; Pdha1: Pyruvate dehydrogenase E1 component subunit alpha; Dlat: Dihydrolipoamide S-acetyltransferase; Aco2: Aconitase 2; Ogdh: Oxoglutarate (alpha-ketoglutarate) dehydrogenase; Sucla2: Succinyl-CoA ligase [ADP-forming] subunit beta; Suclg1: Succinate-CoA ligase, alpha subunit; Suclg2: Succinate-CoA ligase, beta subunit; Slc25a1: Solute carrier family 25, member 1; Slc25a3: Solute carrier family 25 (mitochondrial carrier; phosphate carrier), member 3; Slc25a5: Solute carrier family 25 (mitochondrial carrier; phosphate carrier), member 5. Data are expressed as means ± SEM. * *p* < 0.05 vs. CON; ^†^ *p* < 0.05 vs. ASP; ^§^ *p* < 0.05 vs. GLU.

To determine whether caloric and non-caloric sweeteners might influence mitochondrial fatty acid oxidation and glucose catabolism downstream of glycolysis, i.e., via the TCA cycle, we quantified the levels of 47 mitochondrial proteins involved in fatty acid β-oxidation, the TCA cycle, and the oxidative phosphorylation pathway in total liver protein extracts. In agreement with the PPARα protein expression pattern, a number of enzymes involved in mitochondrial fatty acid β-oxidation were downregulated by glucose but not by other sweeteners. These enzymes include enoyl-CoA hydratase (ECHS1), electron transfer flavoprotein (subunit ETFB), electron transfer flavoprotein-ubiquinone oxidoreductase (ETFDH), and the liver isoform of carnitine palmitoyltransferase 1 (CPT1A) (Figure 6A). The downregulation of these enzymes suggests a degree of suppression of fatty acid oxidation by glucose. However, increased protein concentrations of trifunctional protein subunits HADHA and HADHB may indicate a compensatory response. Fructose and aspartame largely had no effect on protein concentrations of β-oxidation enzymes, except for downregulation of CPT1A by fructose and trifunctional protein subunit HADHA by aspartame, and upregulation of ECHS1 by aspartame (Figure 6A and Table A1).

The proteomics of TCA cycle enzymes showed that both glucose and fructose caused an increase in protein levels of two components of the acetyl-CoA producing pyruvate dehydrogenase complex, i.e., pyruvate dehydrogenase E1 component subunit α (PDHA1) and dihydrolipoyllysine-residue acetyltransferase (E2 component; DLAT), and the effect was more profound in the FRUC group compared with the GLU group (Figure 6B). The protein concentration of the citrate transporter (SLC25A1) was only affected by glucose feeding, but a number of TCA cycle enzymes downstream of citrate were downregulated by both glucose and fructose (Figure 6B), suggesting a negative regulation of TCA cycle activity by both sweeteners. Aspartame feeding largely had no effect on concentrations of TCA cycle enzymes except for downregulation of 2-oxoglutarate dehydrogenase (OGDH) (Figure 6B and Table A1).

The analysis of enzymes involved in the oxidative phosphorylation pathway showed that this pathway was largely unaffected by the sweeteners (Table A1), except for downregulation of phosphate carrier protein (SLC25A3) and adenine nucleotide translocase 2 (SLC25A5) (Figure 6C), indicating decreased supply of substrates for mitochondrial ATP synthesis.

## 4. Discussion

In the present study, we aimed to elucidate the effects of caloric and non-caloric sweeteners on in vivo liver lipid metabolism in rats and to determine their roles in the development of liver steatosis. We showed that fructose and not glucose consumption led to an increase in hepatic lipid content, which was accompanied by an increased conversion of ^13^C-labeled glucose to lipids in the liver as determined in vivo by MRS. Therefore, de novo lipogenesis appears to be an important contributor to fructose-induced liver lipid accumulation, which was confirmed by the strongly increased expression of lipogenic enzymes upon fructose feeding. Aspartame consumption did not significantly affect hepatic lipid content or metabolism, while it similarly reduced whole-body glucose homeostasis compared with glucose and fructose.

The rise in obesity and obesity-related disorders has been associated with the increased consumption of fructose over the past decades [6,7]. In Western diets, fructose is mainly consumed through sugar-sweetened beverages, containing 10–13% (*w*/*v*) of fructose [39]. In rat models, the feeding of fructose both in pelleted diets and in the drinking water has been shown to lead to hypertriglyceridemia [18,40]. However, in contrast to high-fructose pelleted diets that contain 50–70% fructose, diets incorporating 10% (*w*/*v*) of fructose in drinking water have been reported not to modify plasma glucose or insulin concentrations [18]. In the current study, we therefore chose to use 13% (*w*/*v*) of fructose or glucose in the drinking water, which is also better comparable with the consumption of sugar-sweetened beverages by humans. We observed that fructose feeding resulted in significantly elevated plasma TG concentrations when compared with aspartame feeding, whereas this effect was not observed with glucose feeding. Plasma glucose concentrations were not significantly affected, but plasma insulin levels tended to be increased upon the feeding of both glucose and fructose. Therefore, effects of hyperinsulinemia on liver lipid metabolism cannot be excluded, but these effects are expected to be similar for glucose and fructose feeding.

The animals that received the sugar-sweetened drinking water consumed up to 50% less pelleted chow than the animals receiving normal drinking water or the aspartame solution. Nevertheless, the total energy intake in the sugar groups was higher, due to the calories from the sugar in their drinking water. The increased total energy intake in both sugar groups did, however, not lead to a greater gain in body weight compared with the animals receiving normal drinking water or the aspartame solution. This is in agreement with previous studies administering 10% fructose and/or glucose solutions to rats [18,41,42,43] and may be explained by the lower dietary quality of their diets (less protein, micronutrients, fiber, and trace elements) as a result of a lower intake of pelleted chow. Because body weight is not always a good indicator of adiposity, especially in animals, analysis of the body composition by determination of fat pad mass is preferred [44]. In the current study, epididymal and perirenal fat pad weights were higher in the two sugar groups compared with the control and aspartame groups, showing that despite the absence of a significant effect on body weight, glucose and fructose feeding led to increased adiposity.

When comparing between the two sugar groups, the addition of glucose to the drinking water resulted in a higher total energy intake compared with fructose, which was due to a higher consumption of the glucose solution compared with the fructose solution. However, the consumption of pelleted chow was higher in the fructose-fed animals compared with the glucose-fed animals. This observation is in agreement with a previous study in rats, in which the less pronounced reduction in solid food consumption upon fructose feeding was explained by a state of leptin resistance produced by the ingestion of fructose [18]. However, in humans, differential effects of glucose and fructose on ad libitum energy intake have not been observed [45].

Seven weeks of fructose administration resulted in an increased amount of lipids in the liver, whereas the administration of glucose did not significantly affect liver lipid content compared with normal drinking water. This observation is in accordance with previous studies comparing the effects of glucose and fructose on liver lipid accumulation both in rodents [18,46,47] and in humans [48].

In order to determine the cause of liver lipid accumulation upon fructose consumption, ^1^H-(^13^C) MRS was performed combined with the oral administration of ^13^C-labeled lipids, to examine dietary lipid uptake, and with the oral administration of ^13^C-labeled glucose, to determine de novo lipogenesis. Chronic fructose feeding has been shown to lead to an overproduction of intestinal apoB48-containing lipoproteins, which was associated with greater stability of intracellular apoB48 and upregulation of the key enzyme involved in intestinal lipoprotein assembly, microsomal TG transfer protein [19,20,21]. Therefore, chronic fructose consumption may promote the absorption of dietary lipids from the intestine, resulting in an increased influx of diet-derived lipids into the liver. However, 4 h after the administration of ^13^C-labeled lipids, the hepatic incorporation of the ingested dietary lipids was similar among all diet groups. Nunes et al. applied the same MRS-based method to determine dietary lipid uptake in the livers of mice fed with pelleted diets containing 60% glucose or fructose for a period of 8 weeks [47]. Also in this study, the liver lipid pools became equally ^13^C enriched in the glucose- and fructose-fed animals after the ingestion of ^13^C-labeled lipids, but no control group fed with normal chow was included. It was furthermore demonstrated that plasma concentrations of apoB48 and apoB100 were similar between the glucose- and fructose fed groups [47]. Together, these results do not support the hypothesis that an increased influx of dietary lipids into the liver causes fructose-induced hepatic lipid accumulation.

We then investigated the contribution of de novo lipogenesis to hepatic lipid accumulation by administering the rats with ^13^C-labeled glucose for a period of 5 days, after which the conversion of ^13^C-labeled glucose to ^13^C-labeled lipids in the liver was measured with in vivo ^1^H-[^13^C] MRS. We showed that the ^13^C enrichment of the liver lipid pool upon ^13^C-labeled glucose administration was 3.5-fold higher in fructose-fed animals compared with animals receiving normal drinking water, and, moreover, that it was 2.8-fold higher in fructose-compared with glucose-fed animals. Therefore, de novo lipogenesis appears to be an important contributor to fructose-induced liver lipid accumulation.

The stimulation of de novo lipogenesis upon fructose feeding was further investigated by determining the protein levels of lipogenic enzymes, ACC and FAS, and of transcription factors involved in the transcriptional regulation of lipogenic genes, SREBP-1c and ChREBP. Glucose and fructose feeding similarly increased the expression of ChREBP (only significantly for glucose versus normal water) and precursor SREBP-1c in the liver, but the protein levels of mature SREBP-1c were not elevated. However, despite the absence of a clear stimulating effect of fructose on ChREBP and SREBP-1c with respect to glucose, the expression levels of ACC and FAS were 1.6-fold and 1.5-fold higher in fructose- compared with glucose-fed animals, respectively. These results confirm the findings of previous studies using sugar-sweetened drinking water [15,18,49]. A possible explanation for the increased protein levels of ACC and FAS in fructose- compared with glucose-fed animals without differences in ChREBP and SREBP-1c could be that the nuclear fractions of ChREBP and SREBP-1c are responsible for inducing the expression of these lipogenic enzymes [13,14], whereas in the current and other studies, protein levels were determined in whole liver homogenates. Janevski et al. [14] showed that in rats fed with diets containing 60% glucose or 60% fructose, protein levels of ChREBP and SREBP-1c were similar in liver homogenates, but were higher in the nuclear fractions from livers of fructose-fed animals, and that this was associated with increased ACC and FAS gene transcription.

Mitochondrial proteomics provided additional proof for the fructose-induced stimulation of de novo lipogenesis. In livers of both glucose- and fructose-fed animals, two components of the pyruvate dehydrogenase complex were upregulated (PDHA1, DLAT), suggesting increased production of acetyl-CoA [40]. This was in agreement with increased activities of glycolytic enzymes, in particular pyruvate kinase, a phenomenon also reported by others [50,51]. Decreased protein levels of several TCA cycle enzymes (ACO2, OGDH, SUCLA2, and SUCLG1), all of them catalyzing reactions downstream of citrate, suggest that TCA cycle activity might have been limited upon glucose and fructose feeding, favoring the translocation of citrate to the cytosol for lipogenesis [52]. Importantly, most of these effects were more profound in response to fructose compared with glucose.

Decreased mitochondrial fatty acid β-oxidation capacity may contribute to the accumulation of hepatic lipids. The protein levels of transcription factor PPARα, which regulates fatty acid catabolism, was decreased in response to glucose but not fructose feeding. However, Roglans et al. showed that fructose feeding reduces PPARα activity without modifying hepatic PPARα protein levels [18]. In agreement, a downregulation of CPT1A, a rate limiting enzyme in mitochondrial β-oxidation, was observed in livers of both glucose- and fructose-fed animals, indicating decreased mitochondrial acyl-CoA uptake and possibly β-oxidation. However, only glucose also induced upregulation of mitochondrial trifunctional protein (subunits HADHA and HADHB), possibly indicating a compensatory response resulting in lesser suppression of β-oxidation compared to fructose. Furthermore, in the livers of fructose-fed animals, we observed accumulation of the CPT1A inhibitor malonyl-CoA, leading to stronger inhibition of fatty acid β-oxidation compared to glucose [15]. The shift of fatty acids away from oxidation and towards esterification will cause an increase in very-low density lipoprotein (VLDL) secretion, which can explain the increased plasma TG levels in the fructose-fed animals [7,9,53,54]. However, taken together our data suggest that hepatic lipid export was not matched to increased production and decreased oxidation.

The administration of aspartame for 7 weeks did not have a significant effect on food intake or body weight compared with controls receiving normal drinking water. The absence of an effect on body weight is in agreement with a study conducted by Palmnas et al., in which rats received a low dose of aspartame in their ad libitum drinking water (5–7 mg/kg/day) for 8 weeks [31]. Those animals, however, consumed less food and drank more fluid compared to the control animals, which was not observed in the current study. Low dose aspartame consumption in drinking water has been shown to increase fasting blood glucose levels and induce glucose intolerance without an effect on fasting plasma insulin levels in both rats [31] and mice [26]. The effects were attributed to compositional and functional changes in gut microbiota, resulting in increased production of short-chain fatty acids. Short-chain fatty acids can serve as gluconeogenic precursors, possibly contributing to increased hepatic glucose production and thus explaining aspartame’s negative effects on glucose tolerance. In a recent study, however, it was shown that inhibition of the gut enzyme intestinal alkaline phosphatase by aspartame’s breakdown product phenylalanine may explain how aspartame promotes glucose intolerance [30]. In the present study, rats consumed a higher dose of aspartame (280 mg/kg/day) compared to the low dose of 5–7 mg/kg/day reported in [31] and the dose of 123.3 mg/kg/day used in [30]. In contrast to those studies, we did not observe an effect of aspartame on blood glucose levels, but aspartame feeding led to higher fasting plasma insulin levels and trends in higher AUC_i_ and the product of AUC_g_ and AUC_i_ compared with controls. This suggests that higher doses of aspartame may induce compensatory insulin production in the pancreas, resulting in maintenance of normal fasting blood glucose levels and glucose disposal.

To date, the effects of aspartame on liver lipid metabolism have been largely unknown. Exploring the impact of aspartame consumption on hepatic dietary lipid uptake and de novo lipogenesis with ^1^H-[^13^C] MRS revealed no significant effects and total liver lipid content was similar between animals receiving aspartame or normal drinking water. The expression of most of the transcription factors and enzymes involved in lipid metabolism was also not significantly affected by aspartame feeding, corroborating that liver lipid metabolism is largely unaffected by aspartame. Despite of this, aspartame feeding similarly reduced whole-body glucose homeostasis compared with glucose and fructose feeding. In the case of glucose and especially fructose, derangements in liver lipid metabolism are thought to contribute to the development of hepatic insulin resistance [11]. Our results indicate that changes in liver lipid content or metabolism do not play a causative role in aspartame-induced glucose intolerance.

## 5. Conclusions

In conclusion, both glucose and fructose feeding increased adiposity, but only fructose led to higher hepatic lipid levels. The increase in hepatic lipid content upon fructose consumption was not caused by an increased uptake of dietary lipids into the liver, but could be attributed to an increase in de novo lipogenesis and presumably a reduction in fatty acid β-oxidation. The non-caloric sweetener aspartame did not significantly affect liver lipid content or metabolism, while its effects on whole-body glucose homeostasis were comparable to glucose and fructose.

## Figures and Tables

**Table 1:** Animal characteristics.

	CON			GLU			FRUC			ASP		
Start body weight (g)	354	±	6	347	±	3	351	±	3	348	±	3
End body weight (g)	445	±	5	444	±	8	456	±	5	437	±	6
Body weight gain (g)	92	±	4	97	±	6	105	±	4	89	±	4
Food intake (kJ/week)	2300	±	25	1170	±	24 ***^,†††^	1447	±	36 ***^,†††,§§§^	2232	±	35
Food intake (g/week)	180	±	2	91	±	2 ***^,†††^	113	±	3 ***^,†††,§§§^	174	±	3
Drink intake (kJ/week)	NA			1757.9	±	48.7 ^†††^	1274.4	±	44.9 ^†††,§§§^	13.4	±	0.5
Drink intake (mL/week)	177	±	4	808	±	22 ***^,†††^	586	±	21 ***^,†††,§§§^	201	±	7
Total energy intake (kJ/week)	2300	±	25	2927	±	35 ***^,†††^	2721	±	20 ***^,†††,§§§^	2246	±	36
Amount sweetener (g/kg BW/day)	NA			36.87	±	1.25 ^†††^	26.16	±	1.00 ^†††,§§§^	0.28	±	0.01
Epididymal fat (g) (*n* = 36)	5.8	±	0.2	7.5	±	0.4 **^,††^	7.1	±	0.3 *^,†^	5.6	±	0.3
Perirenal fat (g) (*n* = 36)	5.8	±	0.4	9.5	±	0.6 ***^,†††^	9.0	±	0.5 ***^,†††^	5.8	±	0.4
Plasma TG (mM)	1.05	±	0.10	1.06	±	0.10	1.23	±	0.04 ^†^	0.87	±	0.04
Plasma ALT (U/L)	24.94	±	2.35	21.71	±	3.11	51.21	±	8.00 **^,§§^	35.44	±	4.32
*Experimental group 1* (*n = 9 per diet group*)												
Liver weight (g)	10.63	±	0.14	9.97	±	0.16	11.39	±	0.22 ^†,§§^	10.17	±	0.25
Liver glycogen (mg/g ww)	87	±	10	78	±	7	75	±	4	67	±	6
*Experimental group 2* (*n = 6 per diet group*)												
Liver weight (g)	12.92	±	0.16 ^###^	13.08	±	0.64 ^###^	16.25	±	0.59 ***^,†††,§§§,###^	13.23	±	0.21 ^###^
Liver glycogen (mg/g ww)	96	±	3	118	±	8 ^###^	117	±	8 ^###^	115	±	8 ^###^
Liver malonyl-CoA (nmol/g ww)	58.37	±	0.97	62.44	±	2.70	68.05	±	2.32 *^,††^	56.31	±	1.82

Data are expressed as means ± standard error of the mean (SEM) of 15 animals per diet group (unless stated otherwise). Experimental group 1 was sacrificed after an overnight fast, while experimental group 2 was sacrificed in a fed condition. CON, normal water control; GLU, 13% (*w*/*v*) glucose; FRUC, 13% (*w*/*v*) fructose; ASP, 0.4% (*w*/*v*) aspartame; NA, not applicable; BW, body weight; TG, triglycerides; ALT, alanine aminotransferase; ww, wet weight. * *p* < 0.05, ** *p* < 0.01, *** *p* < 0.001 vs. CON; ^†^ *p* < 0.05, ^††^ *p* < 0.01, ^†††^ *p* < 0.001 vs. ASP; ^§§^ *p* < 0.01, ^§§§^ *p* < 0.001 vs. GLU; ^###^ *p* < 0.001 vs. experimental group 1.

**Table 2:** Plasma glucose and insulin concentrations during oral glucose tolerance test (OGTT).

	CON			GLU			FRUC			ASP		
Fasting glucose (mM)	4.87	±	0.21	4.44	±	0.27	4.35	±	0.10	5.22	±	0.40
AUC_g_ (mM·h)	13.67	±	0.39	15.07	±	0.55	14.89	±	0.43	13.87	±	0.46
Fasting insulin (pM)	267	±	64	605	±	97	500	±	81	573	±	61 *
AUC_i_ (pM·h)	454	±	64	576	±	75	614	±	54	562	±	52
AUG_g_·AUC_i_ (mM·h·pM·h)	5462	±	672	8640	±	1151	9274	±	1031 *	7824	±	770

Data are expressed as means ± SEM of nine animals per diet group. CON, normal water control; GLU, 13% (*w*/*v*) glucose; FRUC, 13% (*w*/*v*) fructose; ASP, 0.4% (*w*/*v*) aspartame; AUC_g_, area under the glucose curve; AUC_i_, area under the insulin curve. * *p* < 0.05 vs. CON.

## Appendix A. Experimental Procedures

### Western Blot Analysis

Powdered liver samples were homogenized in ice cold lysis buffer containing 1% Nonidet P-40, 0.5% sodium deoxycholate, 0.1% sodium dodecyl sulfate, 150 mM NaCl, 50 mM Tris, 2 mM NaF, 2 mM Na_3_VO_4_, protease inhibitor cocktail (1:200), pH 8. Homogenates were solubilized for 2 h at 4 °C and centrifuged at 14,000× *g* for 10 min at 4 °C. Equal amounts (30 µg) of total liver protein were resolved with sodium dodecyl sulfate polyacrylamide gel electrophoresis (SDS-PAGE) and transferred to nitrocellulose membranes using Trans-Blot Turbo Midi Nitrocellulose Transfer Packs, and Trans-Blot Turbo Transfer Starter System (Bio-Rad Laboratories Inc., Hercules, CA, USA). After blocking with Tris-buffered saline (TBS) containing 0.1% Tween (TBST) and 5% skim milk powder for 1 h at room temperature, the membranes were incubated overnight at 4 °C with antibodies against acetyl-CoA carboxylase (ACC, 1:1000, cat. No. 3662, Cell Signaling, Danvers, MA, USA), fatty acid synthase (FAS, 1:1000, cat. No. 3180, Cell Signaling), sterol regulatory element-binding protein-1c (SREBP-1c) precursor and cleaved protein (1:1000, cat. No. NB600-582, Novus Biologicals, Littleton, CO, USA), carbohydrate-responsive element-binding protein (ChREBP, 1:1000, cat. No. NB400-135, Novus Biologicals), peroxisome proliferator-activated receptor α (PPARα, 1:1000, cat. No. sc-9000, Santa Cruz Biotechnology), and β-actin (1:5000, cat. No. 2066, Sigma-Aldrich). Next, membranes were washed 3 × 5 min with TBST and incubated with a corresponding horse-radish peroxidase-conjugated secondary antibody for 1 h at room temperature. After the final wash of 3 × 5 min with TBST and 1 × 5 min with TBS, the immunocomplexes were detected using SuperSignal West Dura Extended Duration Substrate (Pierce, Thermo Fisher Scientific Inc., Rockford, IL, USA), visualized using ChemiDoc XRS+ imaging system, and quantified using Image Lab analysis software version 3.0 (Bio-Rad Laboratories Inc., Hercules, CA, USA). All data were normalized to β-actin protein levels and expressed relative to the controls (CON).

**Table A1:** Targeted quantitative mitochondrial proteomics in liver.

Gene Name	Protein Name	CON			GLU			FRUC			ASP		
Fatty acid β-oxidation													
Acaa2	Acetyl-CoA acyltransferase 2	34.2	±	0.7	32.5	±	1.2 ^†^	36.5	±	2.3	39.0	±	1.7
Acadl	Acyl-CoA dehydrogenase, long-chain	38.7	±	1.3	34.9	±	1.6	39.0	±	2.1	41.0	±	1.9
Acads	Acyl-CoA dehydrogenase, short-chain	11.4	±	0.5	10.5	±	0.2	10.9	±	0.3	10.1	±	0.4
Acadvl	Acyl-CoA dehydrogenase, very long-chain	12.5	±	0.6	11.5	±	0.2	10.1	±	0.6 *	11.0	±	0.5
Cpt1a	Carnitine O-palmitoyltransferase 1A	8.0	±	0.2	5.7	±	0.4 **	5.8	±	0.4 **	7.0	±	0.4
Cpt1b	Carnitine O-palmitoyltransferase 1B	1.29	±	0.07	1.96	±	0.19	1.46	±	0.12	1.65	±	0.38
Cpt2	Carnitine palmitoyltransferase 2	6.2	±	0.2	6.8	±	0.3	6.5	±	0.6	5.4	±	0.3
Decr1	2,4-dienoyl CoA reductase 1	7.8	±	0.22	7.4	±	0.3 ^††^	8.3	±	0.7 ^†^	10.0	±	0.2 **
Echs1	Enoyl CoA hydratase, short chain, 1	20.0	±	0.8	16.0	±	0.7 *^,†††^	18.7	±	1.5 ^††^	24.1	±	0.9 *
Eci1	Enoyl-CoA delta isomerase	11.9	±	0.7	11.8	±	0.7	12.0	±	1.0	13.1	±	0.6
Etfa	Electron-transfer-flavoprotein, alpha polypeptide	18.9	±	0.8	15.4	±	1.0 ^††^	19.5	±	1.8	23.3	±	1.3
Etfb	Electron-transfer-flavoprotein, beta polypeptide	25.1	±	0.8	20.1	±	1.0 **^,††^	23.9	±	1.0	25.0	±	1.0
Etfdh	Electron-transferring-flavoprotein dehydrogenase	15.5	±	0.4	13.3	±	0.6 **	14.1	±	0.4	14.6	±	0.3
Hadh	Hydroxyacyl-CoA dehydrogenase	34.4	±	1.1	30.9	±	1.2	32.7	±	1.2	34.0	±	1.2
Hadha	Hydroxyacyl-CoA dehydrogenase/3-ketoacyl-CoA thiolase/enoyl-CoA hydratase (trifunctional protein), alpha subunit	16.1	±	0.3	18.3	±	0.4 *^,†††^	17.1	±	0.6 ^†††^	13.5	±	0.6 **
Hadhb	Hydroxyacyl-CoA dehydrogenase/3-ketoacyl-CoA thiolase/enoyl-CoA hydratase (trifunctional protein), beta subunit	13.6	±	0.4	16.5	±	0.3 **^,†††^	15.3	±	0.5 ^†††^	12.0	±	0.4
Slc25a20	Solute carrier family 25, member 20	4.1	±	0.2	3.1	±	0.3 ^†††^	4.4	±	0.3 ^§^	5.4	±	0.4 *
TCA cycle													
Aco2	Aconitase 2	11.1	±	0.4	9.9	±	0.2 *	11.4	±	0.1 ^§§,†^	10.1	±	0.3
Cs	Citrate synthase	5.40	±	0.13	4.66	±	0.14 ^††^	5.59	±	0.39 ^§^	6.04	±	0.09
Dlat	Dihydrolipoamide S-acetyltransferase	7.1	±	0.2	8.7	±	0.5 *^,††^	10.3	±	0.3 ***^,§§,†††^	6.8	±	0.2
Dld	Dihydrolipoamide dehydrogenase	23.3	±	0.7	21.2	±	1.1	23.3	±	1.1	20.7	±	0.4
Dlst	Dihydrolipoamide S-succinyltransferase (E2 component of 2-oxo-glutarate complex)	13.4	±	0.8	11.7	±	0.6	13.2	±	0.2	13.3	±	0.4
Fh1	Fumarate hydratase 1	14.1	±	0.7	12.1	±	0.6	15.9	±	1.5	16.0	±	1.0
Idh2	Isocitrate dehydrogenase 2 (NADP+)	10.0	±	0.4	9.2	±	0.6	9.2	±	0.2	9.0	±	0.3
Idh3a	Isocitrate dehydrogenase [NAD] subunit α	3.3	±	0.4	3.1	±	0.5	2.7	±	0.2	3.2	±	0.2
Mdh2	Malate dehydrogenase 2	31.6	±	1.0	27.2	±	0.9 ^†^	31.2	±	2.0	33.8	±	1.6
Ogdh	Oxoglutarate (alpha-ketoglutarate) dehydrogenase	6.1	±	0.1	5.3	±	0.2 *	5.7	±	0.2	5.3	±	0.2 *
Pdha1	Pyruvate dehydrogenase E1 component subunit alpha	6.1	±	0.2	7.8	±	0.4 *	9.4	±	0.3 ***^,§,††^	7.3	±	0.5
Pdk1	Pyruvate dehydrogenase kinase, isozyme 1	1.06	±	0.01	1.06	±	0.03 ^††^	1.17	±	0.02 **^,††^	1.05	±	0.02
Slc25a1	Solute carrier family 25, member 1	11.8	±	0.4	10.2	±	0.4 *	11.7	±	0.3	11.6	±	0.5
Slc25a10	Solute carrier family 25, member 10	9.6	±	0.5	6.8	±	0.5 **^,††^	8.0	±	0.4	9.5	±	0.6
Slc25a11	Solute carrier family 25, member 11	5.5	±	0.4	4.6	±	0.2	4.7	±	0.4	5.1	±	0.3
Slc25a22	Solute carrier family 25, member 22	3.99	±	0.11	3.31	±	0.09 **^,†^	3.22	±	0.11 **^,††^	3.85	±	0.15
Sucla2	Succinyl-CoA ligase [ADP-forming] subunit beta	8.2	±	0.3	6.5	±	0.2 ***^,††^	6.9	±	0.2 **	7.8	±	0.3
Suclg1	Succinate-CoA ligase, alpha subunit	18.9	±	0.6	15.2	±	0.5 **^,†^	16.3	±	0.7 *	18.5	±	0.7
Suclg2	Succinate-CoA ligase, beta subunit	11.6	±	0.3	10.3	±	0.3 ^††^	10.9	±	0.2 ^†^	12.7	±	0.6
Oxidative phosphorylation													
Ndufs1	NADH dehydrogenase (ubiquinone) Fe-S protein 1	5.4	±	0.3	4.5	±	0.2 *	5.8	±	0.2 ^§§^	5.1	±	0.2
Sdha	Succinate dehydrogenase complex, subunit A, flavoprotein	10.0	±	0.3	8.9	±	0.3 *	9.7	±	0.2	9.9	±	0.3
Sdhb	Succinate dehydrogenase complex, subunit B, iron sulfur (Ip)	6.8	±	0.2	5.1	±	0.1 *^,†††^	6.9	±	0.5 ^§§^	7.4	±	0.3
Uqcrc2	Ubiquinol-cytochrome c reductase core protein II	8.5	±	0.3	7.3	±	0.3 ^†††^	9.1	±	0.5 ^§^	9.9	±	0.4
Cox5a	Cytochrome c oxidase subunit Va	5.31	±	0.24	4.86	±	0.23 ^††^	5.70	±	0.08 ^§^	5.79	±	0.11
Cycs	Cytochrome c, somatic	3.26	±	0.25	2.94	±	0.14	2.88	±	0.09	3.13	±	0.13
Atp5b	ATP synthase, H+ transporting, mitochondrial F1 complex, beta polypeptide	63.2	±	2.1	58.4	±	2.5 ^†^	71.5	±	5.2 ^§^	73.0	±	2.1
Slc25a3	Solute carrier family 25 (mitochondrial carrier; phosphate carrier), member 3	22.4	±	0.7	17.2	±	0.5 ***	19.3	±	0.9 *	19.0	±	0.8 *
Slc25a4	Solute carrier family 25 (mitochondrial carrier; phosphate carrier), member 4	0.97	±	0.04	0.87	±	0.05	0.86	±	0.02	0.83	±	0.03
Slc25a5	Solute carrier family 25 (mitochondrial carrier; phosphate carrier), member 5	49.4	±	1.7	39.8	±	1.3 **	43.6	±	1.9	42.7	±	1.1 *
Ucp2	Uncoupling protein 2	0.80	±	0.04	0.84	±	0.06	0.70	±	0.04	0.71	±	0.06
Ucp3	Uncoupling protein 3	0.92	±	0.04	1.08	±	0.12 ^†^	0.81	±	0.04	0.78	±	0.05

Data are expressed as means ± SEM of six animals per diet group. CON, normal water control; GLU, 13% (*w*/*v*) glucose; FRUC, 13% (*w*/*v*) fructose; ASP, 0.4% (*w*/*v*) aspartame. * *p* < 0.05, ** *p* < 0.01, *** *p* < 0.001 vs. CON; ^†^ *p* < 0.05, ^††^ *p* < 0.01, ^†††^ *p* < 0.001 vs. ASP; ^§^ *p* < 0.05, ^§§^ *p* < 0.01 vs. GLU.
